# Rapid Analgesia for Prehospital hip Disruption (RAPID): findings from a randomised feasibility study

**DOI:** 10.1186/s40814-019-0454-1

**Published:** 2019-06-12

**Authors:** Jenna K. Jones, Bridie A. Evans, Greg Fegan, Simon Ford, Katy Guy, Sian Jones, Leigh Keen, Ashrafunnesa Khanom, Mirella Longo, Ian Pallister, Nigel Rees, Ian T. Russell, Anne C. Seagrove, Alan Watkins, Helen A. Snooks

**Affiliations:** 1 Swansea University Medical School, Swansea, UK; 2 Swansea Bay University Health Board, Swansea, UK; 3 Patient and public representative, c/o Swansea University Medical School, Swansea, UK; 4 Welsh Ambulance Services NHS Trust, Saint Asaph, UK; 5 Cardiff School of Medicine, Cardiff, UK

**Keywords:** Hip fracture, Fractured neck of femur, Analgesia, Pre-hospital, Paramedic, Fascia iliaca compartment block, Randomised trial, Feasibility study

## Abstract

**Background:**

In managing hip fracture, effective pain relief before admission to hospital is difficult without risking side effects. Although emergency departments routinely use fascia iliaca compartment block (FICB), there has been little evaluation of its use by paramedics before hospital admission. We aimed to assess whether a multi-centre randomised trial to evaluate FICB was feasible.

**Methods:**

Volunteer paramedics used scratchcards to allocate patients with hip fracture at random between FICB and pain relief as usual. Primary outcomes were mortality and quality of life. We also measured adverse events, costs, final diagnosis, length of stay in hospital, pain scores and quality of care and collected qualitative data about acceptability to patients in interviews, and paramedics in focus groups. We pre-specified criteria for deciding whether to progress to a fully powered trial based on the recruitment of paramedics and patients, delivery of FICB, retrieval of outcome data, safety, acceptability, and diagnostic accuracy of hip fracture.

**Results:**

We effectively met all progression criteria: we recruited 19 paramedics who randomly allocated 71 patients between trial arms between 28 June 2016 and 31 July 2017; 57 (31 experimental arm, 26 usual care arm, 80% overall) retrospectively consented to follow-up. Just over half (17/31) of experimental participants received FICB; all others had contraindications, including nine taking anticoagulants. Four of the 31 participants assigned FICB and six of the 26 assigned usual care died within 6 months of hospital admission. Serious adverse events were also similar: 3/35 experimental versus 4/36 in usual care. Paramedics’ recognition of hip fracture had sensitivity of 49/64 (77%) with a positive predictive value of 46/57 (81%). We received quality of life questionnaires for 30 of 49 patients (61%) at 1 month and 12 of 17 (71%) at 6 months. Patient satisfaction was similar: experimental mean 3.4 (*n* = 20) versus 3.5 (*n* = 13) for usual care.

**Conclusions:**

RAPID met all progression criteria within reasonable limits. As equipoise remains, we plan to undertake a fully powered multi-centre trial to test clinical and cost effectiveness of paramedic-administered FICB at the scene of hip fracture.

**Trial registration:**

ISRCTN 60065373 sought 5 November 2015.

**Electronic supplementary material:**

The online version of this article (10.1186/s40814-019-0454-1) contains supplementary material, which is available to authorized users.

## Introduction

### Background

Hip fracture is a common, very painful injury, particularly affecting vulnerable elderly people [1]. Hip fractures generate more admissions to orthopaedic trauma wards than any other injury, and an average inpatient stay of 21 days, thus accounting for 2.5% of all hospital beds [2]. This has a major financial effect on the National Health Service (NHS) [3]. Hip fracture is followed by high short-term mortality − 5% at 30 days, 10% at 6 months, and 20% at 1 year [4–6], and delay to surgery beyond 48 h makes outcomes worse [7–9].

Prehospital management of patients with hip fracture can exacerbate pain as the injury site is difficult to immobilise. Paramedics can administer paracetamol, opioids, and Entonox; intravenous (IV) morphine is most frequently used [10]. Several studies have suggested that prehospital pain relief for patients with suspected hip fracture is inadequate, with up to 40% of patients not receiving any pain relief [11–16]. This shortfall may partly be due to reluctance to administer opioids [17, 18].

Elderly people who sustain hip fractures often have co-morbidities and are vulnerable to the side effects of opioids [19, 20]. These side effects may need ameliorating by further treatments—for instance, naloxone for respiratory depression, laxatives for constipation, or dialysis for opioid accumulation in renal failure. Avoiding opioids in this population may therefore reduce morbidity and length of stay in hospital and improve health-related quality of life [21–27].

Fascia iliaca compartment block (FICB) [28] is increasingly used in emergency departments (EDs) and orthopaedic wards in the care of patients with hip fracture. There is extensive evidence that FICB in-hospital is straightforward to administer, can be performed by non-medical health professionals, and provides adequate pain relief with fewer side effects than opioids [29–47]. Prehospital FICB has been tested twice—in a prospective observational study of 100 patients in the Netherlands [48] and in a randomised trial of 35 patients in Australia [49]. Both studies provided morphine to those receiving FICB. Rapid Analgesia for Prehospital hip Disruption (RAPID) is the first randomised trial of FICB before hospital admission in which one arm avoids opioids.

We hypothesise that the use of FICB to provide prehospital pain relief to patients with a hip fracture, thus avoiding morphine, will improve patient outcomes. In accordance with best practice in evaluating complex interventions [50], we tested the feasibility of delivering FICB and of associated trial methods.

### Aim

To assess the feasibility of undertaking a fully powered, multi-centre randomised controlled trial (RCT) to test the clinical and cost-effectiveness of paramedics providing FICB as early pain relief to patients with hip fracture.

### Objectives

To assess:

1. Willingness of both patients and paramedics to participate in the trial
2. Compliance with the FICB protocol by paramedics
3. Which outcome measures and costs to use in a full RCT and when they should be collected
4. Acceptability of FICB to provide pain relief in the prehospital care of patients with hip fracture
5. Accuracy of recognition of hip fracture by paramedics
6. Sample size required for a fully-powered RCT, the period and number of sites required to recruit these
7. Whether trial processes and outcomes achieve specified progression criteria for progress to fully-powered trial

## Methods

### Trial design

Single-centre randomised parallel-group feasibility trial, with allocation ratio 1:1 and qualitative data collection [51].

### Changes after the study began

1. The Principal Investigator (PI) in the receiving hospital (SF) could provide assent for patients who died before being approached for consent. In the study population, there were likely to be deaths before consent could be sought. As mortality was an outcome of this trial, to exclude patients who died before consent could be sought would mean that the results were not valid for the population. Obtaining consent from a consultee regarding the use of routinely collected healthcare data for research in these circumstances would be distressing. We therefore obtained approval for the local PI to sign an Early Mortality Declaration Form to confirm that the patient has died, and take responsibility for the use of routinely collected healthcare data for this trial.
2. Patients could consent to follow-up of medical data alone, but not to complete questionnaires. This was changed following low consent rates in the first few weeks of the study, and with input from the Paramedic Research Support Officer (PRSO) and Patient and Public Involvement (PPI) members, as we thought some patients would prefer not to complete questionnaires.
3. We included the Quality of Care Monitor in postal questionnaires sent to participants after 1 month instead of when approaching them earlier for consent. We changed this following feedback from the PRSO and PPI members, who indicated that the original approach of completing the questionnaire with the PRSO present might make patients feel uncomfortable.
4. To improve the response rate of self-reported outcome measures, we sent reminders to non-responding participants 2 and 4 weeks after their initial questionnaire.

### Setting

We conducted the trial in the catchment area of one hospital emergency department in Wales.

### Participants

**Fig. 1:**
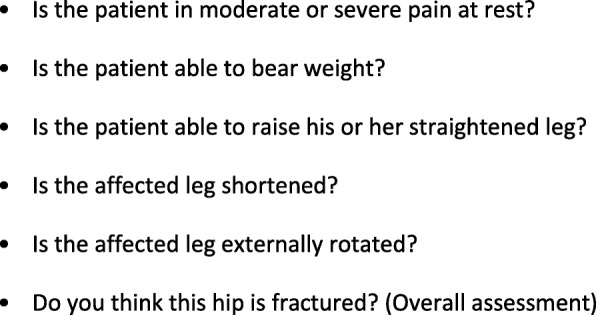
Hip Fracture Assessment Checklist

Inclusion criteria:

Adult patients attended by a participating study paramedic following a 999 call in the catchment area of the receiving hospital and assessed as having an isolated hip fracture, using a checklist developed for the study (Fig. 1); conscious (Glasgow Coma Scale Score of ≥13); and haemodynamically stable.

Exclusion criteria:

Patients who refused analgesia or were combative or attended by a participating paramedic working alone.

### Patient consent

As per normal practice, paramedics sought oral consent to administer analgesia. As it is not ethically appropriate to consent patients to research in medical emergencies [52], the PRSO (LK) sought consent from the patient, or assent from a relative or friend or the hospital PI, to take part in the trial within approximately ten working days of the injury [53–56].

### Data collection

We collected routine data from ambulance service clinical records and hospital notes for each consented participant. We posted questionnaires to patients and enclosed stamped addressed envelopes. We stored all data in REDCap and assured the quality of these data [57].

Appropriate and well-conducted qualitative research can make an important contribution to feasibility studies for randomised controlled trials providing information on acceptability and practical implementation issues [58, 59]. We aimed to interview ten participants who had received FICB—by telephone or in their homes, according to their preference—to explore their experience of receiving FICB and acceptability of the intervention.

Towards the end of the recruitment period, we planned to conduct three focus groups with paramedics in a local ambulance station. We invited all trial paramedics to take part and offered them honoraria to reimburse their time. We sought experiences of receiving training and administering FICB. With participants’ consent, we audio-recorded interviews and focus groups and transcribed these for analysis.

The aim of the health economics was to establish the feasibility of an economic evaluation alongside the main trial. The specific objectives of the health economic component were to identify the relevant NHS and non-NHS resource use to be collected alongside the main trial, to determine acceptability and completeness of resource use and utility measures, and to give preliminary estimates of the NHS costs of the intervention. We estimated intervention training costs using trainers’ hourly pay rates, amortised material and equipment costs, and estimated numbers of patients attended by trained paramedics.

### Interventions

The experimental intervention comprised two elements:

1. Paramedic training

We trained paramedics to perform FICB using an online package, including a video showing the administration of FICB, a 3-h classroom session led by a consultant anaesthetist (SF), and training sessions at the participating hospital to administer FICB to patients. We required paramedics to successfully perform at least three blocks and to critique three blocks before recruiting patients to the study. We offered refresher training at any time during the patient recruitment period.

2. FICB administration

**Fig. 2:**
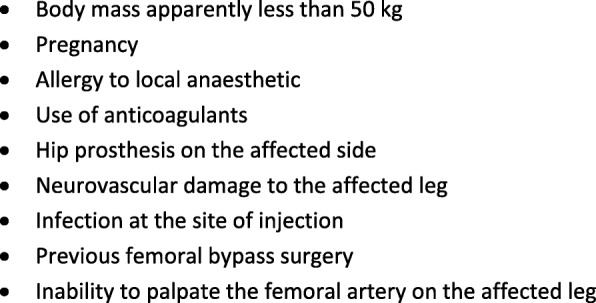
Contraindications to FICB

Paramedics administered FICB after ensuring the patient did not have any of the contraindications in Fig. 2. They could provide the patient with paracetamol and Entonox in addition to FICB, but were asked not to give morphine for at least 20 min after the patient had received the FICB, and then only if the FICB had not relieved the patient’s pain. The detailed protocol for administering FICB, which complies with the TIDieR checklist [60], is in Additional file 1: Table S1.

Usual care:

Patients randomly allocated to the control arm receive usual pain relief care (Entonox, paracetamol and morphine), as judged appropriate by the paramedic.

We collected the following proposed outcome measures for the fully powered trial:

#### Primary

Self-reported health-related quality of life, using SF-12 [35] at 1 and 6 months; mortality within 6 months.

#### Secondary

##### Self-reported:

- Mobility, using the modified Rivermead Mobility Index [36] at 1and 6 months
- Satisfaction with care using a modified Quality of Care Monitor [38] at 1 month
- Qualitative data from patient interviews and paramedic focus groups

##### Routinely collected:

- Length of inpatient stay
- Duration of paramedic management of patient (from paramedic arrival to ED handover)
- Use of anti-emetics and analgesia
- Time between ED handover and surgery
- Pain scores, recorded prior to randomisation by attending paramedic, and on arrival at ED by triage nurse
- Serious adverse events (SAEs) within 7 days of 999 call

Due to time constraints, only those patients recruited in the first 7 months of the trial who were alive were followed up with a 6-month questionnaire.

To assess accuracy of paramedic recognition of hip fracture, the PRSO followed up randomised participants and identified other patients with hip fracture attended by trial paramedics within the period of the trial from hospital records.

### Outcomes

To meet our feasibility objectives, we measured the following outcomes:

a) Number of paramedics who volunteer to take part in the trial
  b) Percentage of randomised patients who consent to follow-up in the trial
Percentage of patients randomly allocated to the experimental group who receive FICB
a) Proportion of data we are able to collect for each proposed primary outcome for a subsequent fully powered trial
  b) Assessment of whether any proposed outcome measures for a subsequent fully powered trial seemed to show significant differences between groups
a) Satisfaction with care by the ambulance service, measure using a Quality of Care Monitor
  b) Safety
Sensitivity and positive predictive value of paramedic recognition of hip fracture
Sample size, number of sites, and recruitment period for fully powered, multicentre trial
Whether the trial meets its progression criteria

At the outset of the feasibility trial, the Trial Management Group (TMG), including the PPI representatives, specified progression criteria mapped to these outcomes, to be met within reasonable limits. These progression criteria were agreed upon by the Trial Steering Committee (TSC).

1. Recruit at least ten paramedics to conduct the trial
2. At least 60% of recruited participants consent to follow-up
3. At least 50% of intervention participants receive FICB
4. Retrieve primary outcomes for at least 70% of consented participants
5. Clinicians are in equipoise about the safety and effectiveness of paramedic-administered FICB
6. Mean participant satisfaction in the experimental arm at least 80% of that in the usual care arm
7. Balance of SAEs between arms
8. Paramedics recognise hip fracture with sensitivity of 75% and positive predictive value of 85%

### Sample size

Formal sample size power calculations were not performed as this was a feasibility study, focussing on estimating parameters for a subsequent fully powered multi-centre trial, rather than on formal testing of hypotheses. Based on the throughput of patients with hip fracture at the participating hospital [53], we judged that recruiting approximately 50 patients to RAPID would be practical and enable us to assess whether the trial met our progression criteria.

### Randomisation

We produced sequentially numbered scratchcards with concealed trial arm allocation before recruiting patients. The trial statistician (GF) produced randomisation schedules that were stratified so that each paramedic received ten scratchcards with five allocations to each arm.

### Blinding

The nature of the intervention prevented us from blinding paramedics and participants to allocation. The second pain score, on arrival at the ED, was obtained from the patient by the triage nurse, who was blinded to the patient’s allocation.

### Analysis

We used descriptive statistics to assess whether we had met our progression criteria. We (GF and JKJ) conducted statistical analysis by treatment allocated using Stata version 15.0. We calculated descriptive statistics only on baseline characteristics, for example, mean age and age range in each group, and number and percentage of female participants in each group. *T* tests and cross tabulations were performed on potential primary and secondary outcomes for the full trial in order to determine if we remained in equipoise as to whether paramedic administered FICB was clinically and cost-effective. We undertook inductive thematic analysis of data from interviews and focus groups. Two researchers (BAE, JKB), the PRSO (LK), and a representative of patients and the public (SJ) separately read and re-read transcripts to identify categories of data. These were discussed collectively [61, 62] and grouped to generate themes. We used NVIVO software to store transcripts and code data. BAE undertook coding, then collated and drafted these findings, for further input and reflection by the group to test and confirm findings [63–65].

Descriptive analyses were used for the health economics. Market and NHS costs were used to determine preliminary estimates of the cost of training paramedics, and two sensitivity analyses were used to check the impact of varying trainers’ grade and amount of paramedic training sessions [66].

### Public and patient involvement

Public and patient representatives contributed to designing, delivering, overseeing, and disseminating the study. We recruited people with experience of hip fracture, as patients or carers, to the TMG and TSC. We provided briefing sessions before all TMG and TSC meetings. We report on these activities in accordance with the GRIPP2 checklist [67].

## Results

**Fig. 3:**
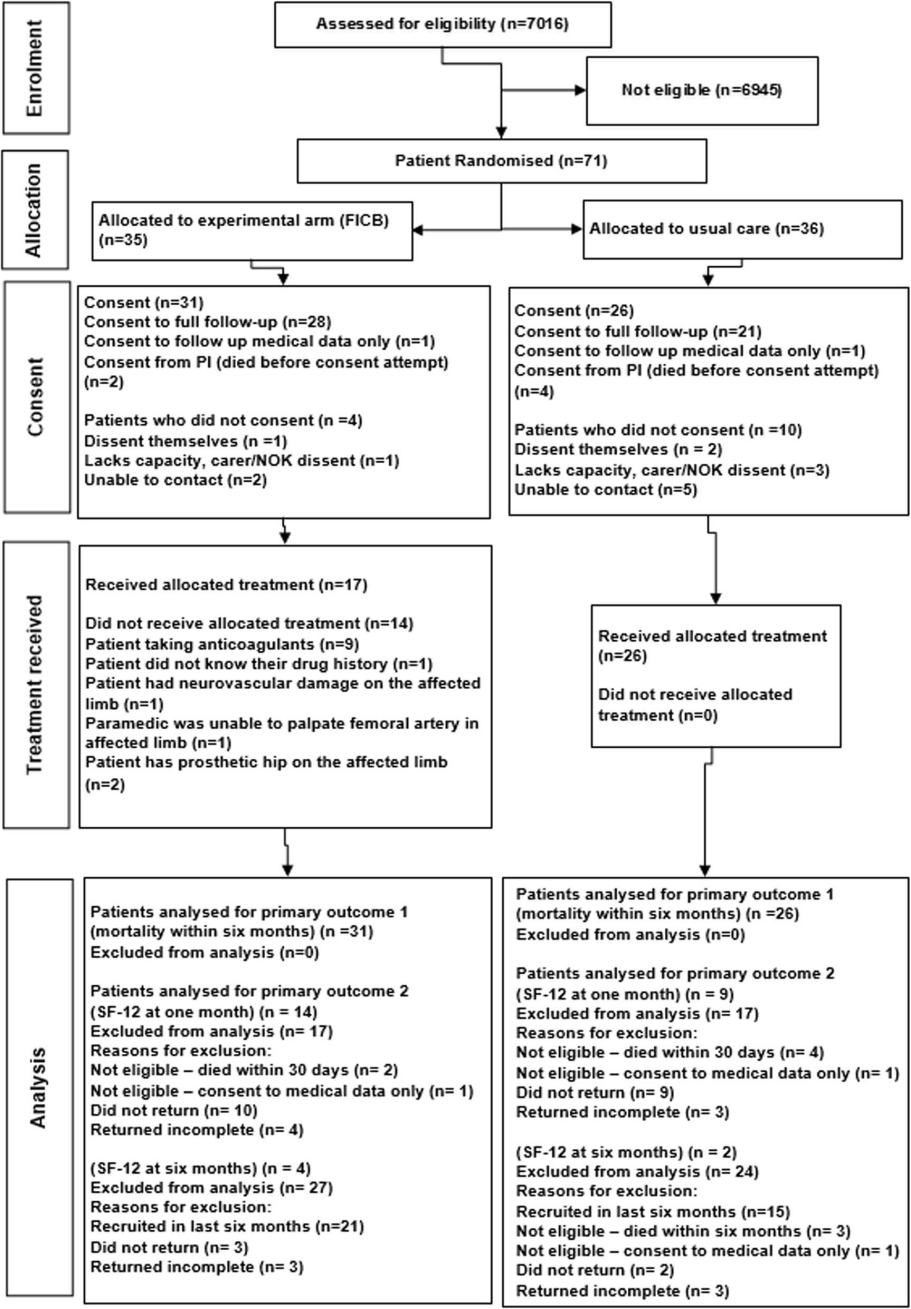
Flow of participants through the trial

We report feasibility study findings in accordance with relevant CONSORT and GRIPP 2-SF checklists [67, 68]; the CONSORT flowchart is seen in Fig. 3.

### Recruitment, consent, and response rates

Nineteen paramedics volunteered and successfully trained to participate, thus achieving Progression Criterion 1. The first 13 trained paramedics recruited participants from 28 June 2016 till 30 June 2017. Six additional paramedics continued recruiting until 31 July 2017 as their training took longer than expected owing to shift patterns; some did not achieve competency until June 2017. Thus, paramedic participation in the trial ranged from 6 to 52 weeks (median 32 weeks). Five of the 19 paramedics did not recruit any patients.

Thirty-one (89%) of 35 experimental participants and 26 of 36 (72%) allocated to usual care consented to follow-up. Thus the overall consent rate was 80%, meeting Progression Criterion 2.

In the experimental arm, 17 of 31 (55%) participants received FICB, meeting Progression Criterion 3. Use of anticoagulants was the most common reason for not using the allocated treatment, reported for nine participants.

Forty-nine participants consented to full follow-up; 30 (61%) participants returned their 1-month questionnaire and 12 of 17 (71%) returned their 6-month questionnaire. We ascertained whether all participants were alive at 6 months. Thus, we met Progression Criterion 4 for two of our proposed primary outcomes, but not for health-related quality of life at 1 month.

### Baseline characteristics

The trial population was elderly (mean age 81.9 years, range 63–101) and mainly female (79%). Trial arms were balanced in age, gender, time of attendance, and attending paramedic at baseline (Table 1).

**Table 1:** Baseline characteristics

	Experimental	Usual care
*N*	31	26
Mean age in years, (range)	81.5, (63.6–101.4)	82.2, (68.3–91.8)
Female, *n* (%)	25 (80.6%)	20 (76.9%)
Time of paramedic attendance		
06:00–17:59 *n* (%)	18 (58.1%)	15 (57.7%)
18:00–05:59 *n* (%)	13 (41.9%)	11 (42.3%)
Attending paramedic ID, *n* (%)		
1	1 (3.2%)	0 (0%)
2	2 (6.5%)	2 (7.7%)
4	3 (9.7%)	1 (3.8%)
5	1 (3.2%)	1 (3.8%)
6	2 (6.5%)	2 (7.7%)
7	5 (16.1%)	4 (15.3%)
8	4 (12.9%)	3 (11.5%)
9	3 (9.7%)	2 (7.7%)
10	2 (6.5%)	4 (15.3%)
12	2 (6.5%)	3 (11.5%)
13	4 (12.9%)	3 (11.5%)
14	0 (0%)	1 (3.8%)
16	1 (3.2%)	0 (0%)
17	1 (3.2%)	0 (0%)

### Outcome analyses

Though it should be noted that this feasibility study was not powered to detect statistically significant differences, we found none between trial arms in unadjusted primary outcomes (Table 2). However, morphine was administered in the experimental arm at approximately half the rate observed in usual care—a statistically significant difference. As we found no other statistically significant differences between arms, notably in satisfaction with care received, we satisfied Progression Criteria 5 and 6.

**Table 2:** Outcomes by trial arm

	Experimental	Usual care	Difference (95% CI)
Primary outcomes			
Mortality/*N*	31	26	
Died within 6 months, *n* (%)	4 (13%)	6 (23%)	-10% (−31% to + 10%)
SF-12 @ 1 month/*N*	14	9	
*Physical Health* mean (SD)	30.1 (7.1)	36.3 (10.2)	− 6.2 (− 13.6 to + 1.4)
*Mental Health* mean (SD)	40.6 (12.9)	34.4 (15.1)	6.2 (− 6.1 to + 18.4)
SF-12 @ 6 months/*N*	4	2	
*Physical Health* mean (SD)	34.2 (10.0)	42.6 (14.9)	− 8.5 (− 36.0 to + 19.0)
*Mental Health* mean (SD)	44.3 (18.4)	57.8 (4.6)	− 13.5 (− 52.2 to + 25.2)
Secondary Outcomes			
Satisfaction with care/*N*	20	13	
Mean (SD)	3.4 (0.4)	3.5 (0.5)	− 0.1 (− 0.4 to + 0.2)
Modified Rivermead Mobility Index			
One month, *N*	18	12	
Mean (SD)	5.2 (3.2)	6.7 (3.3)	− 1.4 (− 3.9 to + 1.0)
Six months, *N*	7	4	
Mean (SD)	8.3 (3.2)	9.8 (2.5)	− 1.5 (− 5.8 to + 2.8)
Pain score, *N*	23	14	
Mean (SD)	3.7 (2.7)	4.1 (2.7)	− 0.4 (− 2.3 to + 1.5)
Medication by paramed/*N*	31	26	
Entonox % (*n*)	3% (1)	4% (1)	− 0.6% (− 10% to + 9%)
Paracetamol % (*n*)	52% (16)	81% (21)	− 29% (− 52% to + 5%)
Morphine % (*n*)	42% (13)	81% (21)	− 39% (− 62% to − 16%)
Ondansetron % (*n*)	35% (11)	23% (6)	+ 12% (− 11% to + 36%)
Time with participant/*N*	31	26	
Mean in minutes (SD)	79.8 (28.2)	74.8 (22.6)	5.0 (− 8.8 to + 18.7)
Time participant in ED/*N*	25	17	
Mean in minutes (SD)	2069 (1694)	2044 (1319)	25 (− 962 to + 1012)
Length of hospital stay/*N*	29	21	
Mean in days (SD)	17.7 (15.2)	26.8 (24.8)	− 9.1 (− 20.5 to 2.3)

The satisfaction with care score is the mean of the final nine question scores; higher scores denote higher satisfaction. The Rivermead Mobility Index score sums the question scores; higher scores denote better mobility

### Harms

Three experimental participants experienced SAEs compared to the four in the usual care arm, thus meeting Progression Criterion 7. Of experimental participants, one experienced local anaesthetic toxicity (successfully treated by the attending paramedic with Intralipid without any indication of long term sequelae), one died within 7 days (from community-acquired pneumonia), and one had sepsis and bowel obstruction (from palliatively treated metastatic cancer). In the usual care arm, one participant had rhabdomyolysis requiring dialysis and intensive care, two patients died within 7 days (from heart failure or pulmonary oedema), and one required a blood transfusion.

### Ancillary analyses

#### Accuracy of recognition of hip fracture by paramedics

From hospital records, we identified 15 ‘false negatives’, i.e. patients eligible for the study, but not recognised as hip fractures by the attending paramedic. Trial paramedics recruited 11 ‘false positives’—participants without radiological evidence of hip or femoral fracture. The resulting sensitivity of 46/61 (75.4%) and positive predictive value of 46/57 (80.7%) thus did not meet Progression Criterion 8, but were within reasonable limits (Table 3).

**Table 3:** Recognition of hip fracture by paramedics

	Hip fracture or femoral fracture on X-ray	No hip or femoral fracture on X-ray	Total
Recruited	46	11	57
Not recruited	15		
Total	61		
Sensitivity (46/61) 75.4%			
PPV (46/57) 80.7%			

We designated three participants with shaft of femur fractures (which FICB would have benefitted) as ‘true positives’ on the advice of the independent TSC. Of the ‘false positives’, three patients had suffered with other fractures—of acetabulum, pelvis, or pubic ramus, but they would not have benefitted from FICB

#### Protocol deviations

We identified 12 protocol deviations (Additional file 2: Table S2). The most common were scratchcards used out of order, once deliberately we suspect, in order to try to reveal an intervention allocation. One experimental participant received morphine before FICB.

#### Health economics

Using year 2017 cost figures, we estimated the cost of an FICB pack to be £19, within the usual range of costs of pain relief. From our single site, we estimated the basic cost of training per patient per year of paramedic activity as £102 with a senior trainer providing initial training, increasing to £170 when the same trainer provides further training. The corresponding costs with a junior trainer were estimated as £88 for initial training and £152 with further training. Though the saving of nine hospital days per participant was not statistically significant, the national cost of non-elective inpatient stays for hip fracture suggest considerable potential for such savings to offset training costs, even by senior trainers with further training, allowing for reasonable geographical variation in training costs.

#### Qualitative results

Eleven paramedics, two female and nine male, took part in three focus groups—one group of five and two groups of three. Three paramedics had been qualified for less than 5 years, three for more than 10 years, and the remaining five for between 5 and 10 years.

Thirteen participants who received FICB consented to interview. When we approached them for interview, one patient who had some cognitive impairment consented to her daughter undertaking the interview instead, since she was present when her mother received FICB. Two patients were too sick to take part, and we could not contact four individuals. In total, we interviewed six patients and one daughter. Interviews took place between 6 and 30 weeks after injury.

Four themes were identified from the focus groups with paramedics: ability and acceptability, patient safety and experience, training, and scope of the paramedic practice [69]. Three themes were identified from patient interviews: memories of receiving pain management and care from ambulance teams, experience of paramedic care, and ongoing hospital treatment and rehabilitation.

Paramedics reported that FICB was a suitable intervention for them to deliver, within their capabilities and in alignment with current practice.

The RAPID trial has just fitted naturally into our everyday pattern… It’s just given us *another* route of pain relief for patients that *definitely* need it. (Focus Group 1 - Paramedic 2)

They were uncertain whether the block effectively reduced patients’ pain, citing examples where it appeared to have made a difference and other instances where it had not. However, they said the drug was potentially better for patients because it reduced the risk of complications from morphine. They reported that it did not change their approach to caring for patients but may have increased the time before patients received pain relief by up to 10 min.

I think it’s a fantastic idea to have (FICB) prehospitally because people die from breaking their hip… they are pumped full of morphine [in hospital] and then they catch a chest infection and they die. It’s something that we can do prehospitally to relieve their pain but also for them to have a more successful outcome. (Focus Group 3 – Paramedic 9)

Respondents praised the training and refresher sessions they received, including the chance to practise with specialists. Suggested ways to improve the training included prehospital scenario-based training, frequent refresher sessions with hospital patients, becoming more familiar with the trial packs, and increasing awareness of the intervention among non-trial staff. Challenges reported included delivering the intervention when family or the public were present, needing to move the patient to administer the injection, being the only trial paramedic on a vehicle, and fearing their skills had decayed when not regularly used. Paramedics supported the use of scratch cards to allocate patients randomly because it was quick and simple and because the card fitted in their pocket.

Participants reported that they had fallen in the house or garden, often when alone, and had waited up to 6 h for an ambulance. Their memories of prehospital care were dominated by their experience of extreme pain, although they did recall the quality of care they received and praised paramedics for their reassuring and calm manner.

I cannot remember exactly what was happening because I was in so much pain. I think somebody gave me something to ease the pain…whatever they did for me, it eased that terrific pain. (Patient 111)

They explained everything – the situation and the reason, did I want to try this and all this. I was glad to see them come in. It was perfect. I could not wish for better. (Patient 78)

Just one respondent recalled the offer of FICB because the paramedic suggested it would enable them to carry him to the ambulance in a chair rather than by stretcher through a window. Participants’ priorities were to reduce the pain of the hip fracture and to regain their mobility and independence. Those who were aware of receiving the block, from what they were subsequently told, said they were happy with the intervention.

#### Sample size required for a full trial

We calculate that 1900 analysable outcomes would suffice to detect a 3-day difference (which we judge to be clinically important) in mean length of inpatient stay (currently 18.5 days) between arms with 90% power and 95% significance level. Our sample size considerations are based around a standardised statistical effect of 0.15, which requires 950 analysable outcomes per arm. Considering the rate of recruitment in this feasibility study (0.1 patient randomised per patient per week), likely dissent rate (10%), and rate of inability to match patients in the Secure Anonymised Information Linkage (SAIL) databank in Wales and NHS Digital in England (1%), we believe study paramedics would need to randomly allocate 2132 patients in total.

The standardised statistical effect is based on the log-Normal distribution (to reflect known skewness in raw LOS), using means and standard deviations which correspond to a difference of 3 days in mean LOS (see above), and reflect feasibility data on SD in LOS (approximately 20 days) in LOS. We supplemented our calculations with extensive simulation experiments to confirm a standardised statistical effect of ~ 0.15 for both raw and logarithmically transformed LOS.

We will assess heterogeneity in LOS between sites through descriptive statistics; if indicated, we will include an appropriate site (fixed or random) effect term in statistical models and use residual diagnostics to determine which yields the better fit.

We envisage that this target would need six collaborating centres each with an average of 40 trained paramedics recruiting patients for 24 months—this takes into account anticipated attrition in study paramedics (due, for example, to sick leave, maternity leave, or career progression). Each site would therefore be expected to recruit approximately 355 patients, though this would vary based upon the different sized catchment areas and population demographics of the receiving hospitals. The recruitment target is thus less than 1% of an estimated total of 237,600 patients estimated to be attended by study paramedics during the recruitment period.

### Public and patient involvement

One of the patient representatives on the TMG attended meetings; the other, unable to travel, contributed through emails and telephone. One patient representative attended TSC meetings. The TMG adopted a patient representative role description describing expectations and available support. The TSC included similar information in its charter.

These patient representatives contributed in several ways in meetings and analysis, including:

- Highlighting consequences of hip fracture and importance of treatment and care
- Advising on obtaining pain scores from injured patients
- Editing patient information sheets to simplify text, reduce content, and add images and space
- Speaking at dissemination events about the impact of hip fracture on vulnerable patients
- Contributing to trial publications as named authors, in particular lay sections.

## Discussion

The RAPID feasibility study met its objectives and achieved all of its progression criteria within reasonable limits (summarised in Table 4). Our findings add to the evidence base that paramedics can administer FICB in prehospital care; whether they should do so remains to be determined.

**Table 4:** Summary of findings by objective and Progression Criterion

Objectives [To assess:]	Relevant Progression Criteria	Relevant result
1. Willingness of both patients and paramedics to participate in the trial.	1. Recruit at least ten paramedics to conduct the trial	19 paramedics took part in the trial
	2. At least 60% of recruited participants consent to follow up	80% of patients consented to follow up
2. Compliance with the FICB protocol by paramedics.	3. At least 50% of intervention participants receive FICB	55% of intervention participants received FICB
3. Which outcome measures and costs to use in a full RCT and when they should be collected	4. Retrieve primary outcomes for at least 70% of consented participants	We checked the mortality status for 100% of participants. 61% of 1 month questionnaires and 71% of 6 month questionnaires were returned
	5. Clinicians are in equipoise about safety and effectiveness of paramedic-administered FICB	
		The only statistically significant difference between arms was the proportion receiving morphine
4. Acceptability of FICB as a method of providing pain relief in the prehospital care of patients with hip fracture.	6. Mean participant satisfaction in the experimental arm at least 80% of that in the usual care arm	Mean participant satisfaction in FICB was 97% of that in the usual care arm
	7. Balance of SAEs between arms	SAEs occurred in three experimental participants and four in the usual care arm
5. Accuracy of recognition of hip fracture by paramedics.	8. Paramedics recognise hip fracture with sensitivity of 75% and positive predictive value of 85%	Paramedics recognised hip fracture with sensitivity 77% and positive predictive value of 81%
6. Sample size required for a fully-powered RCT and period required to recruit these		For example, ~ 1900 patients over 24 months.
7. Whether trial processes and outcomes achieve specified progression criteria for progress to fully-powered trial		We effectively met all progression criteria, with progression criterion four being met for two out of three proposed primary outcomes and progression criterion eight being within reasonable limits.

### Limitations

Of experimental participants, 89% consented to follow-up, compared with 72% of those allocated to usual care; though this difference of 17% is not statistically significant, the full trial will need to guard against, or adjust for, ‘resentful demoralisation’ among control participants.. Though we blinded assessors to the treatments allocated, we could not blind paramedics or patients to the treatment they received, and sham FICB would be unethical.

Paramedics used randomisation scratchcards out of order on four occasions. Although this did not unbalance arms, we shall in future provide scratchcards in a booklet with different fronts and backs so that the cards must be taken in order.

As we stratified randomisation by paramedic, the one who recruited nine participants could have predicted the tenth scratchcard. In future, we shall avoid this risk by using variable block sizes.

This feasibility trial invited paramedics to volunteer; in a full trial, we would need a greater number of paramedics in each site (approximately 40) which may be a challenge. However, other large prehospital trials have recruited significant numbers of paramedics (PARAMEDIC was conducted in four ambulance services and 418 emergency vehicles were recruited—either double-staffed ambulances or single-staffed rapid response vehicles [55]; AIRWAYS-2 was also conducted in four ambulance services and 1523 paramedics were trained [70]). We would monitor recruitment rates closely in the first few months of the trial to ensure the study paramedics are engaged with the trial and make any necessary modifications to improve recruitment rates.

We do not know why some paramedics did not recruit any patients: some may have been those not fully trained until late into the recruitment period; they may have had sick or annual leave; or been working on cars (alone) and therefore ineligible to recruit patients. We plan to monitor for paramedics who do not recruit patients for 3 months or more in the full trial, so that they can be offered refresher training to ensure they still feel confident enough to recruit and randomly allocate patients to trial arms.

### Patient and public involvement

Most patients who suffer hip fractures are elderly and frail and may have dementia. Hence, recruiting patient representatives with relevant experience was challenging [71]. Although we welcomed email and telephone contributions from one TMG member who could not travel to meetings, this left the other representative alone at meetings. This reinforces agreed best practice which recommends a minimum of two representatives at meetings and flexibility in accessing their contributions [72, 73]. In a full multi-centre trial, we will seek public and patient involvement at strategic, site, and local level to reduce the problems for individuals travelling to meetings and to increase the number and roles of representatives. We will also ensure adequate support plus a named contact individual for public and patient members involved in future research [74].

### Interpretation and conclusion

We compared outcomes between trial arms only to confirm that we remain in equipoise about the clinical effectiveness of pre-hospital FICB for hip fracture administered by paramedics (i.e. that there were no stark differences between groups noted in a study not powered to detect this). Hence, we treat observed differences in outcomes with caution in this feasibility trial.

The addition of the health economic perspective alongside this feasibility will improve the efficiency of the main trial. In line with the FORGE trial taskforce guidelines [75], data collection forms were designed and tested in order to capture the NHS resource usage relevant in addressing the research questions, e.g. the time paramedics spent with patients. Missing data is a big issue for health economic evaluation alongside multicentre trials; we would therefore propose to collect resource data from routinely collected databased in the multi-centre trial (i.e. SAIL and NHS Digital).

As responses to questionnaires only just met our progression criterion at the 6-month time point and did not meet our criterion at the 1-month time point, health-related quality of life may not be the best primary outcome in a full trial without modification. For example, we would need to increase the emphasis on telephone follow-up, as our 6-month questionnaire return rate was boosted by this means in the feasibility study, and we would use incentives, which have been proven to improve response rates [76, 77]. Furthermore, the sample size required to detect a plausible difference in mortality would be infeasibly high. Length of inpatient stay is available for all patients and showed a clinically relevant, though statistically insignificant, difference between arms within the feasibility study. We shall therefore consider this as the primary clinical outcome of the definitive trial.

This feasibility study generally met its pre-determined progression criteria within reasonable limits; an application has therefore been prepared and submitted for funding for a fully powered multi-centre randomised trial of paramedic-administered FICB. This application will take into account factors that we have learnt from this feasibility study, for example, allocating enough time for training, providing refresher training, and making non-trial staff aware of the trial. As the positive predictive value of paramedic recruitment of hip fracture failed to reach our pre-specified progression criterion, we propose to monitor for false-positive recruitments during the fully powered trial so that we can ensure any paramedics that need further training in the recognition of hip fracture are identified as early as possible.

## Additional files

Additional file 1: Paramedic pathway to perform a landmark guided fascia iliaca compartment block as part of the RAPID Trial. (DOCX 362 kb)

Additional file 2: Protocol deviations in the RAPID feasibility study. (DOCX 15 kb)

## Abbreviations

AE: Adverse event
ED: Emergency department
FICB: Fascia iliaca compartment block
IV: Intravenous
NHS: National Health Service
PI: Principal Investigator
PPI: Patient and Public Involvement
PRSO: Paramedic Research Support Officer
RCT: Randomised controlled trial
SAE: Serious adverse event
SAIL: Secure Anonymised Information Linkage
TMG: Trial Management Group
TSC: Trial Steering Committee
WAST: Welsh Ambulance Services NHS Trust

## Authors’ information

JKJ is a junior doctor and research officer at Swansea University, undertaking a PhD on prehospital analgesia for patients with hip fracture. AK, ACS and BAE are research officers at Swansea University. JKJ, AK and ACS are the trial managers for RAPID. GF is Professor of Clinical Trials and Director of Swansea Trials Unit. SF is Consultant Anaesthetist in Swansea. KG is Consultant of Emergency Medicine in Swansea. SJ represents patients and the public on the Trial Management Group. LK is the Paramedic Research Support Officer for RAPID. IP is Professor of Trauma and Orthopaedics in Swansea. NR is Head of Research and Innovation for the Welsh Ambulance Services NHS Trust and Chief Investigator for RAPID. ITR is Emeritus Professor of Clinical Trials at Swansea University. HAS is Professor of Health Services Research at Swansea University. AW is Associate Professor of Statistics in Swansea Trials Unit.
