# Supplementary material for: Rapid Analgesia for Prehospital hip Disruption (RAPID): findings from a randomised feasibility study
Source: Pilot Feasibility Stud. 2019 Jun 12;5:77. doi: 10.1186/s40814-019-0454-1 (PMC6560881; doi:10.1186/s40814-019-0454-1)
Supplement: Supplementary file 1 — Paramedic pathway to perform a landmark guided fascia iliaca compartment block as part of the RAPID Trial. (DOCX 362 kb) [file 40814_2019_454_MOESM1_ESM.docx]

**Paramedic Pathway to Perform a Landmark Guided**

**Fascia Iliaca Compartment Block as part of the RAPID Trial**

| **TASK** | **RATIONALE** |
| --- | --- |
| 1. Confirm indication for Fascia Iliaca Compartment Block [FICB] (using hip fracture assessment checklist tool) | To ensure inclusion criteria are met |
| 1. Explain the procedure and potential side effects of an FICB to the patient and carer/next-of-kin and ensure consent is taken (verbal)   Include: - Permanent nerve damage [very rare]   - - Local anaesthetic toxicity [very rare]   - Local bruising, infection and bleeding [uncommon]   - Failure of blockade [5-20%] | To ensure the patient makes informed consent about receiving FICB |
| 1. Establish monitoring using Dinamap^®^ (non-invasive blood pressure, pulse oximetry) | To facilitate recognition of any potential adverse events |
| 1. Record patient’s vital signs and pain score before providing analgesia. If the patient has cognitive impairment and is unable to verbalise a pain score, please provide a subjective pain score, and note on the patient clinical record that you have done so. | To provide baseline observations and assess efficacy of FICB |
| 1. Confirm patent intravenous access | To facilitate treatment of any potential adverse events |
| 1. Ensure that resuscitation equipment and emergency drugs are readily available before commencing FICB | To facilitate treatment of any potential adverse outcomes |
| 1. Assist the patient into the supine position with affected leg extended as much as possible. | To provide correct position of leg for FICB |
| 1. Undertake physical examination of patient’s lower limb. Confirm pulse in foot. | To detect FICB contraindications |
| 1. Check correct side against patient’s notes and verbal confirmation if possible   **‘STOP BEFORE YOU BLOCK’** | To confirm the correct leg is to be blocked |
| 1. Place one middle finger on Anterior Superior Iliac Spine [ASIS] and the other middle finger on the symphysis pubis. Divide the line using both index fingers into three equal parts as shown   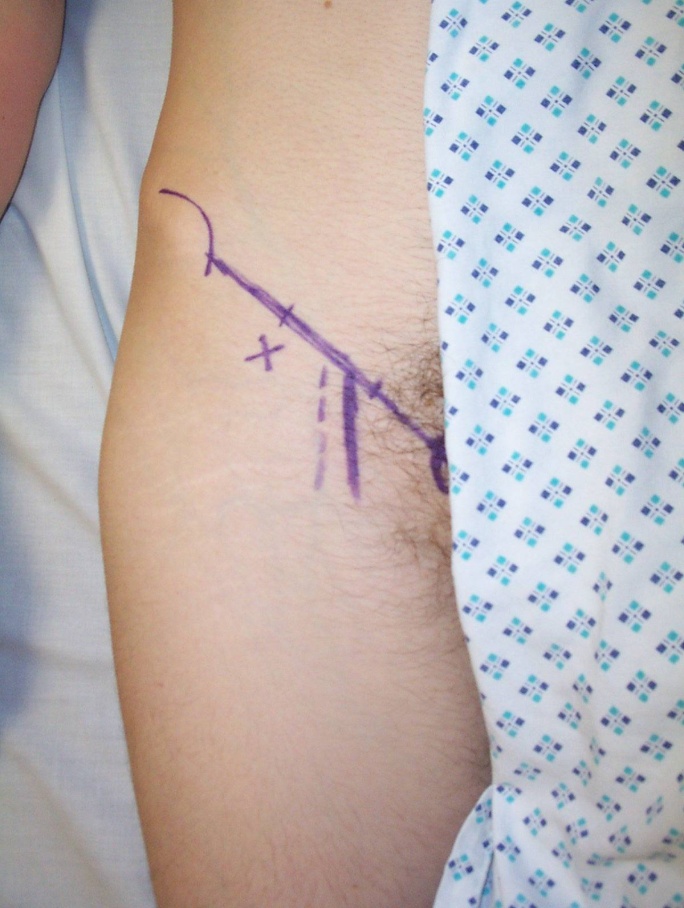 | To ensure accurate needle placement |
| 1. Mark the injection point 1cm below the lateral index finger | To facilitate block placement |
| 1. Confirm the femoral artery position is medial to the injection point (palpate femoral artery making sure it is at least 2cm medial to marked injection point)   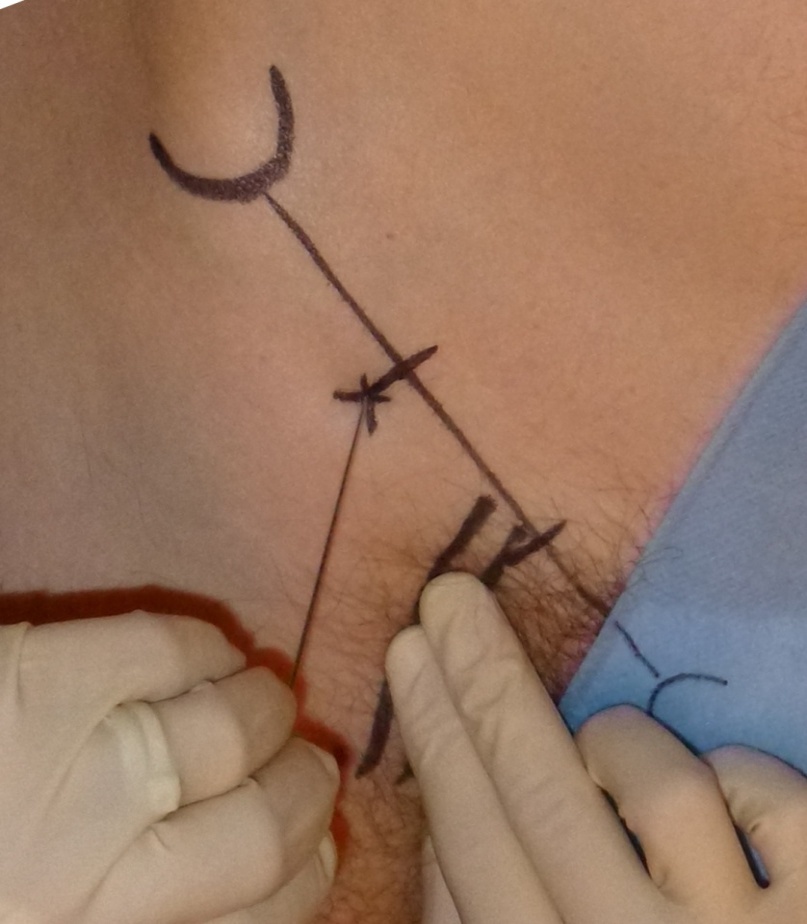 | To avoid complications |
| 1. Wash hands, prepare equipment and sterile field:  - Open dressing pack, syringes, needles, local anaesthetic etc - Clean the skin from the ASIS to the pubic bone with provided skin preparation - Wash hands - Put on sterile gloves - Draw up prilocaine 1% into 1 x 20ml and 1 x 10ml syringe and flush the extension line ensuring Bsmart pressure monitor positioned between syringe and needle. | To minimise the risk of introducing infection |
| 1. Insert the 18G block needle perpendicular to the skin at the marked point  - **Do not aim needle medially** | To prevent accidental damage / injection of the femoral nerve |
| 1. Advance the needle  - Advance through **two distinct pops** [loss of resistance felt following penetration of Fascia Lata and Fascia Iliaca] - If pop not clear, bring needle back under skin and slightly change angle - Once in position, the needle can be released | To ascertain correct placement of under the Fascia Iliacus muscle compartment |
| 1. Aspirate and if no blood detected:  - Slowly inject Prilocaine 1% x 20ml total aspirating every 5 mls - There should be no resistance to the injection. The Bsmart monitor should not progress into the yellow zone; if there is, pull back cannula slightly and retry injection   Correct placement is confirmed by:   - No resistance to injection - No appearance of subcutaneous swelling - Onset of analgesia over 20-minutes | To reduce the risk of accidental intravenous injection and ensure correct placement of Prilocaine |
| 1. Change syringe and complete Prilocaine injection according to patient weight (total dose 30ml if >50kg body weight). Remove cannula | To prevent overdose of local anaesthetic |
| 1. Monitor and record patient observations over first 30-minutes   **NB** **The biggest risk of local anaesthetic toxicity is during the first 20 minutes following bolus administration** | Early detection of adverse effects |
| 1. Discard all sharps and used equipment, in accordance with WAST policy, in the appropriate containers i.e.  - Sharps box - Clinical waste bin | To minimise sharps injury  To allow for the correct disposal of possible contaminated equipment |
| 1. Instruct the patient to inform the paramedic crew if they experience any tingling/numbness around the mouth, tinnitus and/or if they feel ‘strange’ | Local anaesthetic toxicity or hypersensitivity reaction may occur after Prilocaine injection |
| 1. Record the procedure on the trial documentation provided, indicating any problems or complications encountered and action taken | To ensure accurate records are maintained and adverse events can be investigated |
| 1. Advise the patient not to mobilise without the assistance of a staff member | Blockade of the motor nerves of leg may occur, limiting ability to mobilise safely |
| 1. When the patient is handed over to emergency department staff, please remind the triage nurse to take a pain score from the patient | To assess efficacy of the FICB |
