# Supplementary material for: Rapid Analgesia for Prehospital hip Disruption (RAPID): findings from a randomised feasibility study
Source: Pilot Feasibility Stud. 2019 Jun 12;5:77. doi: 10.1186/s40814-019-0454-1 (PMC6560881; doi:10.1186/s40814-019-0454-1)
Supplement: Supplementary file 2 — Protocol deviations in the RAPID feasibility study. (DOCX 15 kb) [file 40814_2019_454_MOESM2_ESM.docx]

|  | **Deviation** |
| --- | --- |
| 1 | Morphine administered before FICB |
| 2 | Scratchcard selected at random instead of in order – in attempt to get intervention |
| 3 | Out of date Prilocaine 1% used because paramedic did not check trial pack before giving to patient (no harm came to the patient) |
| 4, 6, 7 | Paramedic used scratchcard from back of pack instead of front (accident) |
| 5 | Paramedic randomised patient with suspected hip fracture who was also having a stroke – to intervention, but did not have time to give FICB |
| 8 | Patient not approached for consent as they were on the End of Life Care Pathway |
| 9 | Patient with a distracting injury randomised inappropriately |
| 10 | Association of Anaesthetists of Great Britain and Ireland (AAGBI) protocol for administration of Intralipid followed instead of RAPID protocol when both documents were in intralipid pack |
| 11 | Date of randomisation not written on the scratchcard and scratchcard not signed |
| 12 | Paramedic administered FICB to patient in ED at the request of ED staff – after patient recruitment had ended. |

Protocol deviations in the RAPID feasibility study
